# A simple scoring of beam walking performance after spinal cord injury in mice

**DOI:** 10.1371/journal.pone.0272233

**Published:** 2022-08-11

**Authors:** Shunsuke Ito, Yohei Kakuta, Kosuke Yoshida, Yuma Shirota, Tokue Mieda, Yoichi Iizuka, Hirotaka Chikuda, Haku Iizuka, Kazuhiro Nakamura

**Affiliations:** 1 Department of Orthopedic Surgery, Gunma University Graduate School of Medicine, Maebashi, Gunma, Japan; 2 Department of Laboratory Sciences, Gunma University Graduate School of Health Sciences, Maebashi, Gunma, Japan; 3 Department of Orthopaedic Surgery, Isesaki Municipal Hospital, Isesaki, Gunma, Japan; Uniformed Services University, UNITED STATES

## Abstract

Precise evaluation of motor functions using simple and reproducible tests for mouse models of spinal cord injury (SCI) are required. Overground walking of SCI mice has been tested by Basso Mouse Scale for locomotion (BMS). In contrast, only a few works quantify walking performances of SCI mice on narrow beams, a different task. Here, we established a novel scoring system using a single beam walking apparatus for SCI mice. The scoring system uses binary judgments of values such as retention, moving forward and reaching the goal on a beam for rating. In addition, high score was given to SCI mouse when the mouse efficiently used hindlimbs for locomotion on the beam. A high rate of concordance of the score derived from positions of hindlimbs between two observers was obtained. Mice displayed the lowest total score on the beam immediately after the SCI, then the score gradually increased like time course of BMS score. Furthermore, the total scores reflected gradation of severity of SCI in 2 strains of mice. The beam walking score proved to be strongly correlated with that of BMS score, indicating that performances between overground walking and beam walking are partly correlated in SCI mice. Collectively, the novel scoring system offers an opportunity to easily evaluate motor performances of mice with SCI.

## Introduction

Spinal cord injury (SCI) is a devastating major trauma that results in dysfunctions of the motor, sensory and bladder systems [1]. For SCI patients, the severity of SCI is determined during the recovery period as well as the acute stage immediately after the injury. As for the motor symptoms long after SCI, a retrospective study showed rare complete motor recovery even after surgical or conservative treatment [2]. Likewise, only 9% of SCI patients turned out to gain full recovery at discharge [3]. These studies indicate that deficits in motor functions seem to be persistent in majority of SCI patients and therefore, precise evaluation of multiple motor functions is needed for long time after SCI.

Mouse SCI shares behavioral and histological abnormalities with human SCI. Motor behavioral tests using qualitative parameters is needed for elaborate evaluation of SCI animals. One of the most established behavioral test that uses gait pattern on ground as parameters to detect motor dysfunctions of SCI mice is the Basso Mouse Scale for locomotion (BMS) [4]. The parameters used for the BMS scoring include ankle movement, frequency of plantar stepping, paws position, trunk stability and tail position [4]. The use of these parameters were justified by the observation that majority of mice revealed recovery of plantar stepping and trunk instability significantly after SCI [4]. Similar scoring system for locomotion of rats in open field named the Basso-Beattie-Bresnahan scoring (BBB) was also established earlier [5].

On the other hand, walking on a narrow beam is a motor task probably requiring abilities that are partly different from overground walking. Beam-walking test that uses quantitative values such as walking distance, number of slips and time elapsed to reach the goal on the narrow beam is suitable to detect motor incoordination [6]. It was suggested that the test has higher sensitivity than the rotarod test in detecting deficits in motor coordination [7]. The beam-walking apparatus was used for the cortical trauma as well [8]. For rat SCI, the beam-walking apparatus has been used to collect objective parameters [9–11] although qualitative parameters were also used in some works [12, 13].

For mice, the beam walking apparatus was also used in SCI and in focal demyelination in the white matter of spinal cord in a few papers [14–17]. In these works, multiple beams with different widths were used for scoring and various protocols for the scoring were used.

In the present investigation, we aimed at establishing a simple and reproducible behavioral measure using a single beam walking apparatus for SCI mice. To this end, we searched for simple parameters that require binary judgments for scoring and found that retention, moving forward and reaching the goal on the beam were associated with BMS scores. In addition, positions of hindlimbs on a beam was also scored, which had high inter-rater agreement. Using the all parameters above, the total score of SCI mouse on the beam was obtained. The total scores of 2 mouse strains reflected gradations of SCI severity and were highly associated with BMS scores.

## Materials and methods

### Mice

Mice with genetic background of ICR and C57BL/6 were maintained in specific pathogen-free room. The temperature in the room was kept at 23°C and lighting was 12 h every day. Animal experiments were approved by the Animal Resource Committees of Gunma University. We followed NIH guidelines to treat mice and made every effort to minimize the suffering of the mice and the number of mice used for the experiments. Mice were randomly divided into SCI groups with different severity. Twelve SCI mice (6 males and 6 females of the 2 strains) were used for each SCI group (mild and severe SCI of ICR and C57BL/6 mice). The sham groups consisted of 5 mice each (2 males and 3 females of ICR mice and 3 males and 2 females of C57BL/6 mice). The ages of these mice were 9–13 weeks old and the body weights ranged from 29 g to 47 g in ICR and from 19 g to 27 g in C57BL/6 mice.

### Spinal cord contusion injury

SCI was produced essentially as described [18]. Briefly, a laminectomy was done after anesthesia with ketamine and xylazine (100 mg/15 mg/kg, respectively, i.p.). The device to contuse the exposed spinal cord around T10 was an Impactor model III spinal cord contusion system (W. M. Keck Center for Collaborative Neuroscience, The State University of New Jersey). A 5.6 g rod with an impact head 1 mm in diameter was placed 4.25 mm above the T10 spinal cord and dropped to cause mild injury (n = 12 for both ICR and C57BL/6 mice), whereas heights of 10.5 mm were applied for severe injury (n = 12 for both ICR and C57BL/6 mice). The severity was different from our previous paper in which the rod was placed 6.25 and 12.5 mm above the T10 spinal cord for mild and severe SCI, respectively [19]. The wounds were sutured using nylon thread after the SCI. We massaged the lower abdomen of SCI mice to prevent bladder and rectal disorder. No infection was recognized during observation period.

### BMS

The scoring protocol of BMS is precisely described in a previous literature [4]. Briefly, each mouse was separately placed in an open field (60 cm x 120 cm) and ankle movement, plantar stepping, paw positions, tail position, trunk instability and coordination were visually examined during spontaneous walking in the open field for 2 min. Averaged values were collected when the score was different between the right and left hindlimbs. The scoring was done by an observer using recorded video before SCI and 1 day, 1 week, 2 weeks, 3 weeks and 4 weeks after SCI.

### Scoring system using beam-walking apparatus

The apparatus of the beam-walking test consists of a round horizontal bar 100 cm long and 11 mm in diameter and was elevated 50 cm. The beam was made of steel having coarse surface. A black box was attached to one end of the bar. The goal point was 10 cm before the black box. The start point was the opposite side and 10 cm in from the end of the bar. Therefore, the distance between the start point and the goal point on the beam was 80 cm. Before the SCI, mice were thoroughly trained to traverse the narrow beam from the start point to the opposite black box until the mice reach the goal twice without stopping. The tests were done in the room having a strong light.

One point each was given to the mouse with retention, moving forward, reaching the goal and slipping frequency of less than 60% on the beam during observation of 2 minutes. The slipping frequency was determined by counting the number of slips of hindlimbs and total steps. The mouse which cannot take steps using hindlimbs did not fulfill the criteria of slipping frequency of less than 60% and therefore yielded 0 point. Regarding position of hindlimbs on the beam, the mouse which did not use hindlimbs got 0 point. When the mouse used the hindlimbs, the mouse got 1, 2 or 3 point; sandwiching the beam with thighs, sandwiching the beam with plantar and putting plantar on the beam resulted in 1, 2 and 3 points, respectively. The point values for all the parameters were summated. Therefore, the total score was from 0 to 7. We tested 3 times and the highest point was used for each parameter to calculate the total score. The scores were obtained before SCI and 1 day, 1 week, 2 weeks, 3 weeks and 4 weeks after SCI.

### Statistical analysis

The values were expressed as the mean ± SE. We used non-parametric tests because data did not reveal normal distribution. Statistical significances between two groups were analyzed by Mann-Whitney U test, whereas those among three and four groups were examined using Kruskal-Wallis test with Steel-Dwass analysis. For repeated measures, Friedman test followed by Scheffe test was applied as previously described [20]. Inter-rater agreement was estimated by kappa statistic. Correlation between the values of different behavioral tests was calculated by Spearman’s rank correlation coefficient. *p* values less than 0.05 were defined as statistically significant.

## Results

### Association of locomotor performances on a narrow beam with BMS scores of SCI mice

We tried to establish a novel scoring using a single beam walking apparatus for SCI mice. Owing to paralysis of both hindlimbs, acute SCI mice show forelimb walking instead of quadrupedal walking. Because such acute mice cannot stay on the beam for long, evaluation using only time to reach the goal and number of slips may not be suitable for these mice. Therefore, we searched for the parameters on the narrow beam that are closely associated with severity of SCI. Since the BMS score reliably reflects severity of SCI, we decided to use the parameters that affect BMS score.

**Fig 1:**
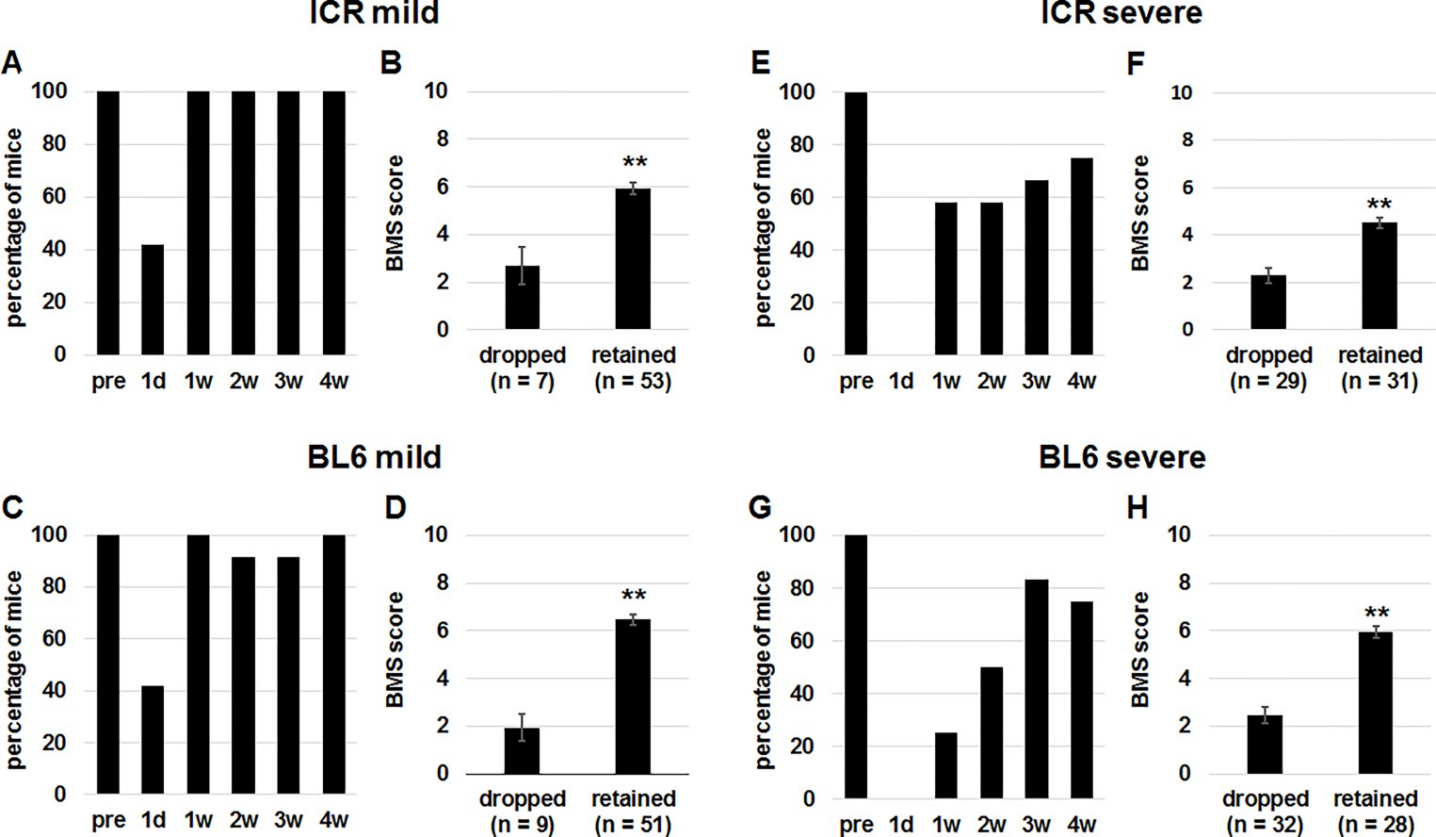
Comparison of BMS scores between mice which fell down from the beam and those which retained on the beam after SCI. (A, C, E, G) Percentages of ICR (A, E) and C57BL/6 (C, G) mice before (pre) and 1 day (d), and 1, 2, 3 and 4 weeks (w) after mild (A, C) and severe (E, G) SCI which did not fall down from the beam during the test. (B, D, F, H) ICR (B, F) and C57BL/6 (D, H) mice 1 day, and 1, 2, 3 and 4 weeks after mild (B, D) and severe (F, H) SCI were divided into those which dropped from the beam and those which retained on the beam and BMS scores of the 2 groups were compared. Mann-Whitney U test, **p < 0.01.

Mice immediately after SCI fail to traverse the beam. Even motor function of these mice can be evaluated using retention on the beam. Before the mild SCI, percentages of ICR (Fig 1A) and C57BL/6 (Fig 1C) mice that retained on the beam were 100%. One day after SCI, the percentages declined to approximately 40% in the two strains (Fig 1A and 1C). Then, the percentages returned to 100% from 1 week after SCI (Fig 1A and 1C). In the ICR and C57BL/6 mice 1 day to 4 weeks after SCI, only 12–15% of mice could not retain on the beam (Fig 1B and 1D). When BMS scores of the two groups were compared, the BMS scores of retained mice were significantly higher in the 2 strains of mice (Fig 1B and 1D). In contrast to the mild SCI, percentages of mice that retained on the beam were 0% one day after severe SCI in both ICR (Fig 1E) and C57BL/6 (Fig 1G) mice, and the percentages did not reach 100% even at 4 weeks after SCI (Fig 1E and 1G). From 1 day to 4 weeks after severe SCI, around 50% of SCI mice failed to retain on the beam (Fig 1F and 1H). Notably, BMS scores of mice 1 day to 4 weeks after severe SCI were significantly higher in the retained group than the dropped group in both ICR (Fig 1F) and C57BL/6 (Fig 1H) mice. Taken together, mice that could retain on the beam essentially showed higher BMS scores and therefore, we decided to use the parameter for the scoring.

**Fig 2:**
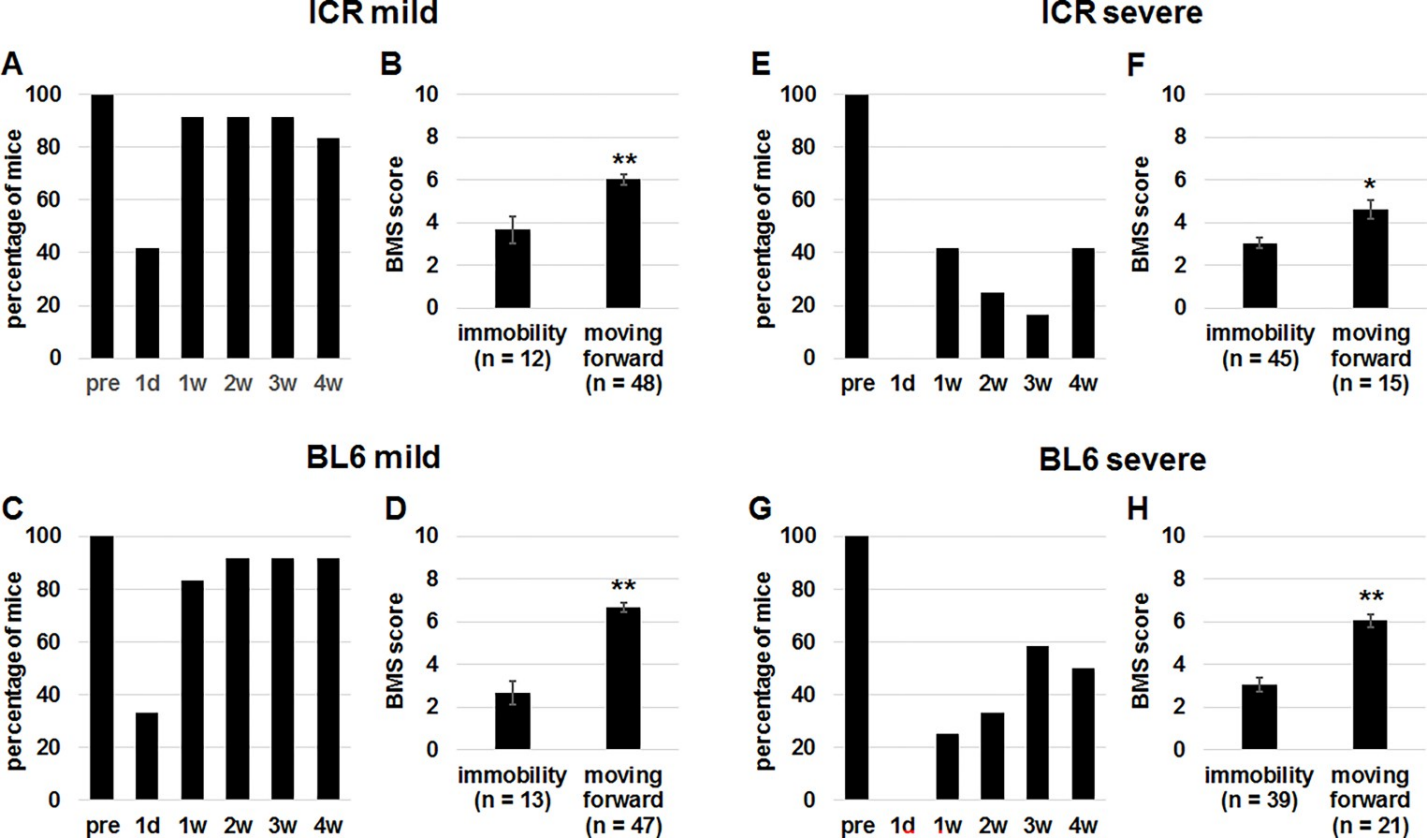
Comparison of BMS scores between mice which did not move forward on the beam and those which did after SCI. (A, C, E, G) Percentages of ICR (A, E) and C57BL/6 (C, G) mice before (pre) and 1 day (d), and 1, 2, 3 and 4 weeks (w) after mild (A, C) and severe (E, G) SCI which moved forward on the beam during the test. (B, D, F, H) ICR (B, F) and C57BL/6 (D, H) mice 1 day, and 1, 2, 3 and 4 weeks after mild (B, D) and severe (F, H) SCI were divided into those which did not move forward on the beam (immobility) and those which did. BMS scores of the 2 groups were compared. Mann-Whitney U test, *p < 0.05, **p < 0.01.

The second parameter for mice which failed to traverse the beam is “moving forward” on the beam. The “moving forward” was accomplished when at least 1 forelimb took one step. Before the mild SCI, 100% of ICR (Fig 2A) and C57BL/6 (Fig 2C) mice moved forward on the beam. However, the percentages were less than 50% one day after SCI in the two strains (Fig 2A and 2C). Then, the percentages became more than 80% from 1 week after SCI (Fig 1A and 1C). Approximately 80% of mice 1 to 4 weeks after mild SCI could move forward on the beam (Fig 2B and 2D). The BMS scores of mice that moved forward were significantly higher than those which cannot move in SCI mice of ICR (Fig 2B) and C57BL/6 (Fig 2D) strains. As we expected, percentages of mice that moved forward on the beam were 0% one day after severe SCI in both ICR (Fig 2E) and C57BL/6 (Fig 2G) mice, and the percentages were approximately 40–50% even at 4 weeks after SCI (Fig 2E and 2G). In the SCI mice from 1 day to 4 weeks after severe SCI, only 25% (ICR) and 35% (C57BL/6) of mice could move forward on the beam (Fig 2F and 2H). Remarkably, BMS scores of ICR (Fig 2F) and C57BL/6 (Fig 2H) mice 1 day to 4 weeks after severe SCI which successfully moved forward were significantly higher than those of the failed groups. Collectively, “moving forward” on the beam was essentially correlated with higher BMS scores in multiple severity and strains, which allowed us to add the parameter for the scoring.

**Fig 3:**
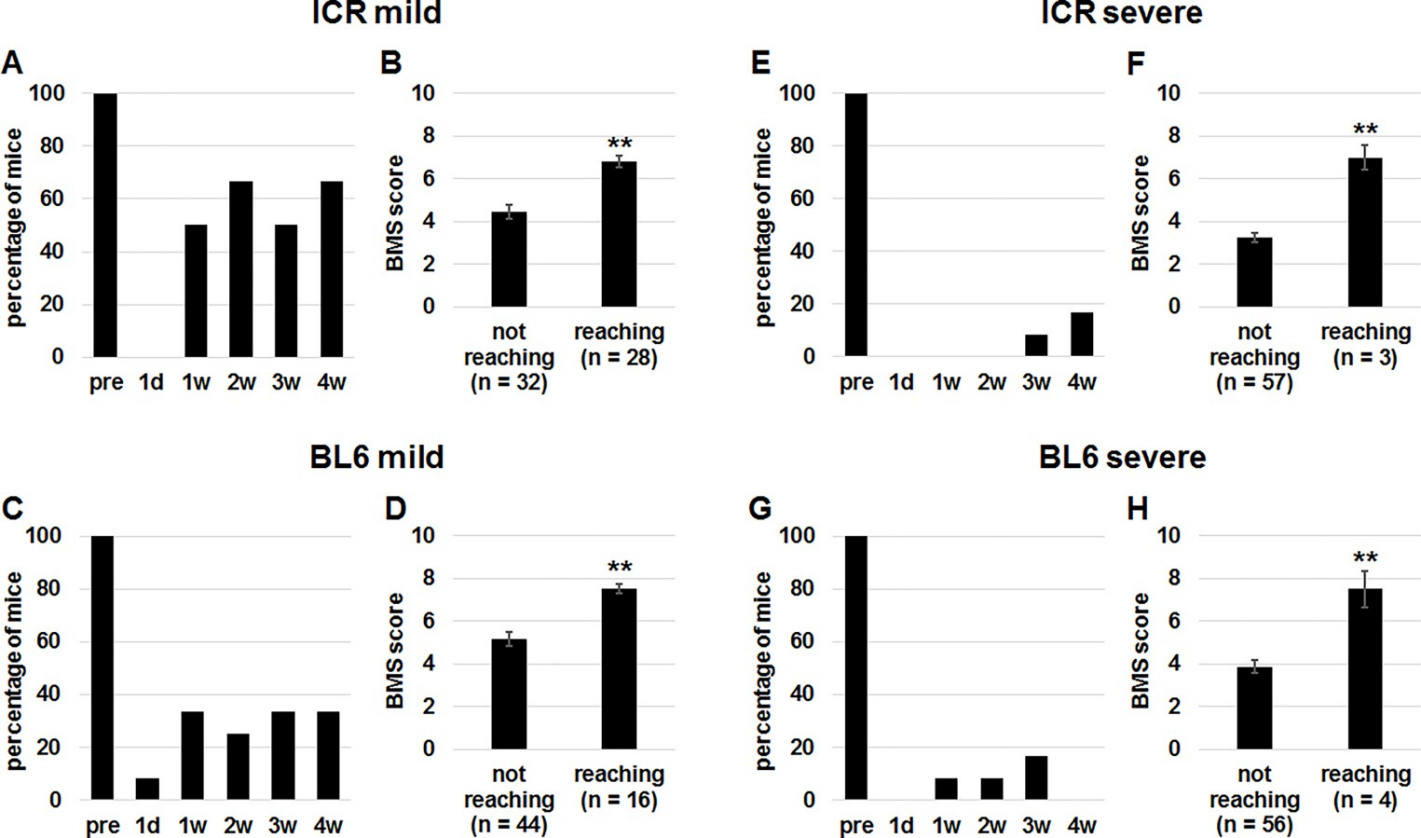
Comparison of BMS scores between mice which did not reach the goal and those which did after SCI. (A, C, E, G) Percentages of ICR (A, E) and C57BL/6 (C, G) mice before (pre) and 1 day (d), and 1, 2, 3 and 4 weeks (w) after mild (A, C) and severe (E, G) SCI which reached the goal during the test. (B, D, F, H) ICR (B, F) and C57BL/6 (D, H) mice 1 day, and 1, 2, 3 and 4 weeks after mild (B, D) and severe (F, H) SCI were divided into those which did not reach the goal and those which did. BMS scores of the 2 groups were compared. Mann-Whitney U test, **p < 0.01.

Although mice immediately after SCI could not reach the goal on the beam, long time recovery period after SCI might make it possible to reach the goal. All mice without SCI could reach the goal (Fig 3A, 3C, 3E and 3G). One day after mild and severe SCI, no mice successfully reached the goal in the two strains (Fig 3A, 3C, 3E and 3G). The percentages of mice that succeeded in reaching the goal were not 100% even in mice of ICR (Fig 3A) and C57BL/6 (Fig 3C) strains 1 to 4 weeks after mild SCI.

Less than 50% of mild SCI mice reached the goal (Fig 3B and 3D), and only 5% of severe SCI mice could reach the goal (Fig 3F and 3H). BMS scores of the mild SCI mice which reached the goal were significantly higher in ICR (Fig 3B) and C57BL/6 (Fig 3D) mice. Likewise, the scores were higher in severe SCI mice of ICR (Fig 3F) and C57BL/6 (Fig 3H) that reached the goal. The fact that the mice which could reach the goal had higher BMS scores motivated us to add the parameter for the scoring.

**Fig 4:**
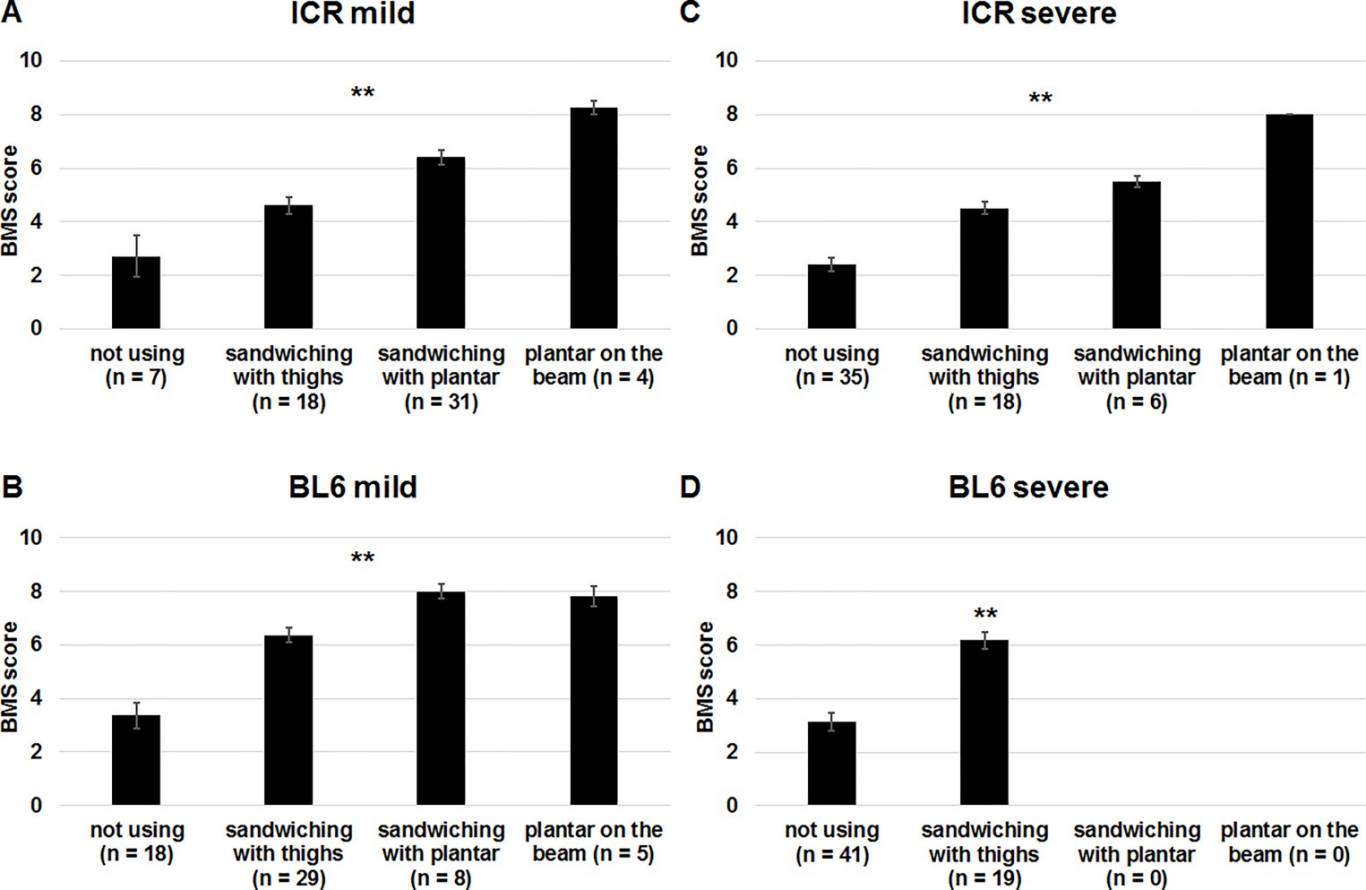
Comparison of BMS scores among mice with different positions of hindlimbs on the beam after SCI. ICR (A, C) and C57BL/6 (B, D) mice 1 day, and 1, 2, 3 and 4 weeks after mild (A, B) and severe (C, D) SCI were divided into 4 groups according to positions of hindlimbs; (i) mice which could not use hindlimbs, (ii) those which sandwiched the beam from the both sides with thighs, (iii) those which sandwiched the beam from the both sides with plantar, (iv) those which put plantar on top of the beam. BMS scores of the groups were compared. Kruskal-Wallis test with Steel-Dwass analysis or Mann-Whitney U test, **p < 0.01.

**Fig 5:**
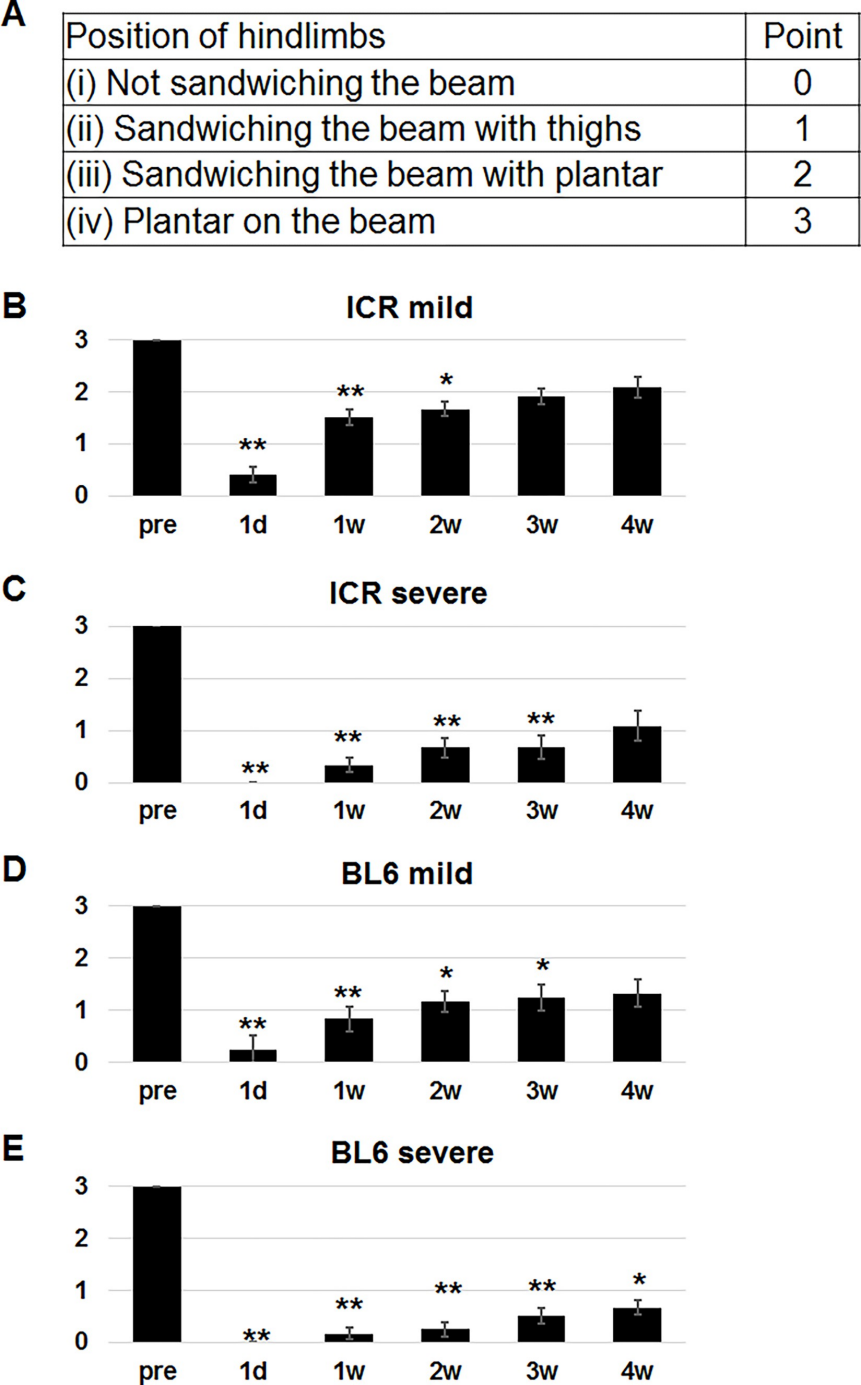
Scoring based on positions of hindlimbs on the beam after SCI. (A) Scoring protocol. (B-E) The scores of ICR (B, C) and C57BL/6 (D, E) mice before (pre) and 1 day (d), and 1, 2, 3 and 4 weeks (w) after mild (B, D) and severe (C, E) SCI. Friedman test followed by Scheffe test was applied for comparison of the scores at each time point after SCI with those before SCI. *p < 0.05, **p < 0.01.

During acute phase of T10 SCI, hindlimbs are paralyzed and are not put on the beam. We then scored position of hindlimbs of SCI mice on the beam. After visual inspection, we noticed that there are 4 patterns as the appearance of hindlimbs on the beam. The first pattern is the case when the mouse does not use hindlimbs for locomotion on the beam. In the second pattern, the mouse uses the hindlimbs by sandwiching the beam with thighs to move forward. In the third pattern, the mouse sandwiches the beam with plantar. The last pattern is that the mouse put plantar on the beam, which most effectively contributes to locomotion on the beam. The mild SCI mice that did not use hindlimbs were only 12% and 30% in ICR (Fig 4A) and C57BL/6 (Fig 4B) mice, respectively. However, these were 58% and 68% in severe SCI mice of ICR (Fig 4C) and C57BL/6 (Fig 4D) strains, respectively. Eight percent of mild SCI mice of C57BL/6 strain put plantar on the beam (Fig 4B). In contrast, no severe SCI mice of C57BL/6 strain could sandwich the beam with plantar or put plantar on the beam (Fig 4D). As shown in Fig 4, there were significant differences in BMS scores among the groups in mild and severe SCI mice of ICR and C57BL/6 strains. The lowest and highest BMS scores were seen in mice that did not use hindlimbs and those putting plantar on the beam, respectively. Therefore, we gave 0, 1, 2 and 3 points for mice not using hindlimbs (S1 Video), those sandwiching the beam with thighs of both sides (S2 Video), those sandwiching the beam with plantar of both sides (S3 Video) and those putting plantar of both sides on the beam (S4 Video), respectively (Fig 5A). When the position of hindlimb of one side was different from that of the other side, we gave the lower score. For example, if one side of plantar was on the beam and the other side sandwiched the beam with thigh, the mouse got 1 point. If one side sandwiched the beam with plantar, whereas the other side sandwiched with thigh, the mouse got 1 point.

Then, the scores of ICR and C57BL/6 strains derived from position of hindlimbs were compared among different time points after mild and severe SCI (Fig 5B–5E). Before SCI, all mice got 3 points. One day after SCI, the points were less than 1.0. Friedman test revealed significant differences over time points for all 4 groups. The significant differences were seen at 1 day, 1 week, 2 weeks and/or 3 weeks after SCI compared to those before SCI in ICR with mild SCI (Fig 5B), ICR with severe SCI (Fig 5C) and C57BL/6 with mild SCI (Fig 5D) groups. Scheffe test revealed that the differences between the scores before SCI and those at 4 weeks after SCI were not significant in the 3 groups, suggesting that position of hindlimbs at 4 weeks after SCI returned to the pattern before SCI in the 3 groups. In contrast, the difference between the score before SCI and that at 4 weeks after SCI was still significant in C57BL/6 with severe SCI (Fig 5E). Therefore, pattern of position of hindlimbs did not return to that before SCI at 4 weeks after SCI in the group. The scoring of the position of hindlimbs is derived from visual inspection. Therefore, different observers might potentially give different scores. To assess reproducibility of the score by different observers, the scores of 48 mice of ICR and C57BL/6 strains at 4 weeks after SCI were given by two observers. As shown in S1 Fig, the scores were correlated between the two observers (Kappa statistic, 0.71; p < 0.001).

**Fig 6:**
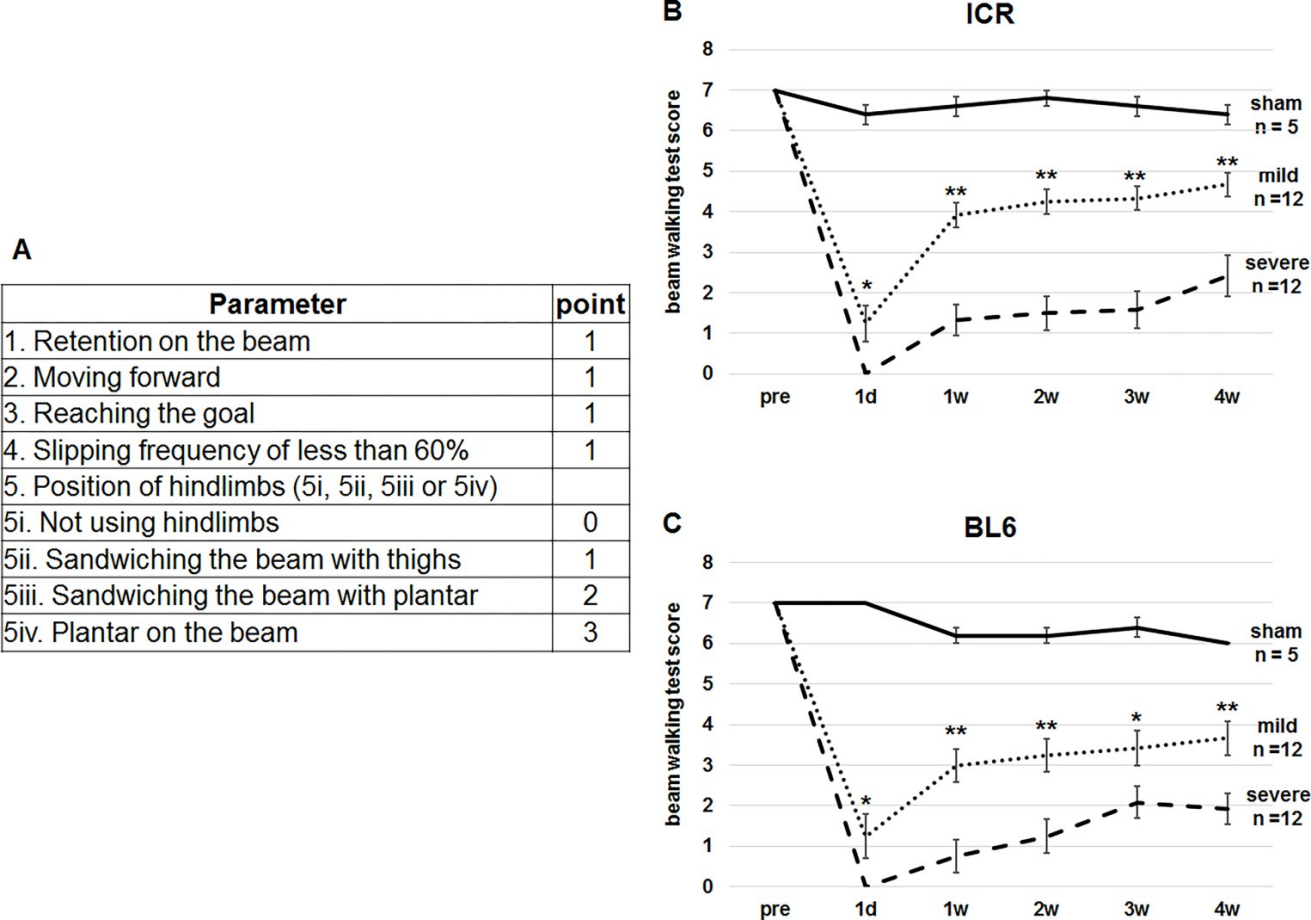
Beam-walking test scoring protocol. (A) The scoring protocol. One point each was given when the mice retained on the beam, moved forward, reached the goal and slipped with frequency of less than 60%. Regarding the appearance of hindlimbs on the beam, 0, 1, 2 or 3 points was given to mice which could not use hindlimbs, those which sandwiched the beam from the both sides with thighs, those which sandwiched the beam from the both sides with plantar and those which put plantar on top of the beam, respectively. Therefore, the total points ranges from 0 to 7. (B, C) The scores of ICR (B) and C57BL/6 (C) mice were compared between mild SCI and severe SCI 1 day (d), and 1, 2, 3 and 4 weeks (w) after SCI. Mann-Whitney U test was used to compare the score at each time point. *p < 0.05, **p < 0.01.

For the total score of SCI mice on the narrow beam, we also gave 1 point each to mice with retention, moving forward and reaching the goal. In addition, number of slips was also included for the scoring because that is an important parameter of motor incoordination [21]. Discrimination between valid step (S4 Video) and slip (S5 Video) was defined as the foot coming off the top of the beam according to a previous literature [22]. Since maximum slipping frequency before SCI was 60%, 1 point was given to mice with slipping frequency of less than 60% on the beam (Fig 6A). The point values for all the parameters were summated. Thus, the total score is from 0 to 7.

### Mouse beam-walking test scores of different SCI severity

To assess whether the novel scoring system for beam walking reflects the degree of severity of SCI, we prepared mild and severe SCI mice. Behavioral scoring was performed before and 1 day after SCI and every week from 1 to 4 weeks after the SCI. Before SCI, mice got maximum score (7 points). Then, all mice with mild and severe SCI showed lowest scores at 1 day after SCI in ICR (Fig 6B) and C57BL/6 strains (Fig 6C). Then, the scores gradually increased. Friedman test revealed significant differences over time points in mild and severe SCI of ICR (Fig 6B) and C57BL/6 (Fig 6C) strains. The significantly lower scores were detected using Scheffe test at 1 day, 1 week, 2 weeks and/or 3 weeks after SCI, as compared with those before SCI for the 4 groups. However, the significant differences were not detected at later time points after SCI for the 4 groups. These results imply that the scoring system can detect recovery of motor functions after SCI.

Comparison of the scores between mild and severe SCI using Mann-Whitney U test clarified significant differences at all time points after SCI in both ICR (Fig 6B) and C57BL/6 (Fig 6C) mice. These results suggest that the novel scoring system accurately distinguishes the performances of mild SCI mice from that of severe SCI in multiple strains of mice. As a control, sham mice got almost maximum scores at all time points in ICR (Fig 6B) and C57BL/6 (Fig 6C) mice. Thus, we decided to apply the current scoring protocol (Fig 6A).

**Fig 7:**
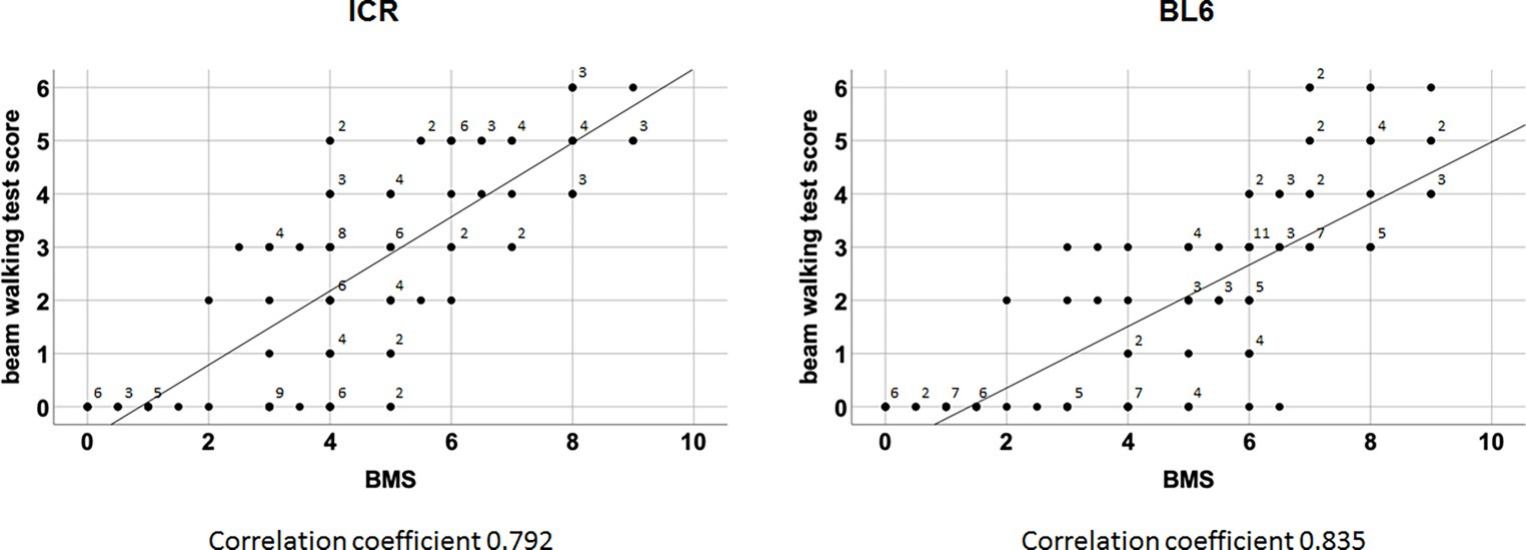
Correlation between the beam walking test score and BMS score. The scores of BMS (x-axis) and the beam walking test (y-axis) from mice 1 day and 1, 2, 3 and 4 weeks after both mild and severe SCI were plotted in the graph. When there are multiple mice showing same beam walking score and BMS score, the corresponding dots were labeled with the numbers of mice. Spearman’s rank correlation coefficient was calculated.

Finally, we checked whether the novel beam walking test score is related to BMS score in SCI mice. The two scoring were done in same SCI mice from 1 day to 4 weeks after mild and severe SCI of ICR and C57BL/6 mice. As shown in Fig 7, the two scores were highly correlated (Spearman’s rank correlation coefficient, 0.792 and 0.835 for ICR and C57BL/6 mice, respectively; p < 0.001). Thus, performances between beam walking and overground walking were correlated in SCI mice.

## Discussion

Refinement of protocols of behavioral tests for SCI enables evaluation of motor functions in different situations. For example, the BBB scoring was optimized to apply to SCI mice [23]. Likewise, a modified scoring was developed by combining the BMS with modified ladder climb and modified grip walk with high predictive values [24]. Importantly, the combined method provided better separation across levels of SCI and also less variability compared to the individual tests [24].

Regarding the present investigation, mice with a same BMS score showed multiple beam walking scores and vice versa (Fig 7). It is plausible to predict that beam walking and overground walking partly require different sets of coordinated muscle contraction. However, given the correlation in the scores of the two tests, same types of coordination were also used for the two motor tasks. Our novel and simple scoring system used retention, moving forward, slipping frequency, reaching the goal and position of hindlimbs on a beam as rating parameters. The scores reflected severity gradations of SCI, which was shared by ICR and C57BL/6 mice. Thus, the scoring can be commonly used for SCI mice.

Although recovery of SCI mice differs from that of SCI rat [4], several works on beam walking tests of SCI rats are informative for the test using SCI mouse. Using qualitative parameters, the locomotion performance of rats on the flat-beam test was scored on a scale from 0 to 7 points [12], on the basis of a paper by Metz and Whishaw [13]. The test evaluated how the animal walked on the beam, for example, by dragging its hind legs or by using one or both hind legs. Likewise, using objective parameters such as the foot-stepping angle and the rump-height index, another research team measured the beam-walking performance of rats using video recording [10]. These parameters correlated with lesion volume, and significant differences were seen between moderately and severely injured rats at 1–9 weeks after SCI.

In contrast, there are only a few papers using beam walking apparatus for mice with SCI or spinal cord demyelination. Using qualitative parameters such as weight supporting steps and dragging and sweeping hindlimb on the beams with width of 1.2 cm or 2.0 cm and 30 cm long, the scores ranging from 0 to 7 were determined for SCI mice [17]. In the present study, we aimed to use parameters determined by binary judgment for SCI mice.

We found that significant numbers of SCI mice could not retain on the beam. When the scoring was made using the 25 cm long beams with 2, 1.6, 1.2, 0.8, and 0.4 cm widths, and the number of misstep and the narrowest width the mice successfully traverse on the beam were recorded after lysolecithin-induced focal demyelination in the white matter of spinal cord, reliable data was obtained [15]. Likewise, using metal beams with similar length (24.13 cm long) and widths (2, 1.6, 1.2, 0.8, 0.4 cm), the number of errors on the beams and the shortest width the SCI mice succeed to traverse were incorporated into a scoring system [16]. A significant recovery of the motor performance over time was obtained in the work. In contrast, when the test was done using longer steel beam (50 cm long) with similar widths (from 2 to 0.5 cm), only a small number of mice could walk on even the widest 2 cm bar at 14 days after moderate compression SCI [14]. Therefore, the length of the beam was found to largely affect the results of beam walking test in SCI mice. In the current study, only few mice with severe SCI reached the goal. We used only 1 size of beam apparatus (1.1 cm in diameter and 80 cm long). The length of beam (80 cm) might be hard for SCI mice to traverse the beam.

We searched for parameters by which the data can be obtained regardless of successful traverse. A candidate was a binary judgment of either forelimb walking or quadruped walking. However, we gave up to use the parameter because majority of SCI mice exhibited forelimb walking on the beam especially during acute phase after the injury. Furthermore, significant percentages of mice several weeks after SCI still exhibited forelimb walking on the beam. Trunk stability likely contributes to the score because that is particularly required for the beam walking of mice with forelimb walking.

Notably, some mice that showed forelimb walking without putting their hindlimbs on top of the beam were using hindlimbs differently to move forward. Their thighs or plantar was found to contact to lateral sides of the beam. Therefore, we divided the mice into 4 groups according to the position of hindlimbs; mice not using hindlimbs, those sandwiching the beam with thighs, those sandwiching the beam with plantar and those putting plantar on the beam. Since we expected that the mice putting their hindlimbs on the beam have highest motor ability, highest score was given to these mice. When we compared BMS scores of the 4 groups of mice, the lowest and highest scores were essentially found in mice not using hindlimbs and those putting plantar on the beam, respectively, and the scores of mice sandwiching the beam were intermediate. These results justified the rating.

Regarding judgment method of beam walking test, a previous paper used an automated device to collect accurate data from SCI animals [25]. In the TreadScan system, which used a transparent treadmill belt, hind limb swing time, stride length, toe spread and track width were automatically measured using a high-speed camera [25]. By contrast, our simple scoring system using a beam requires only a simple and cheap apparatus.

As a common index to evaluate the severity of symptoms in SCI patients, the American Spinal Injury Association (ASIA) impairment scale has been widely used to simply and precisely determine the severity of SCI. However, a more detailed scoring system was recently reported for SCI patients. The novel scoring system, called the Nutech functional score, included multiple critical parameters that are not included in the ASIA scale [26]. The authors suggested that the score is useful to evaluate recovery after treatment in SCI patients [26]. When we consider potential application of our novel scoring system to SCI patients in future, results of beam walking test of patients other than SCI are informative. When the beam walking test was applied to humans with limb loss, Sawers and Ting quantified the ratio of distance walked to total possible distance on a simple beam walking task using experts, novices and individuals with transtibial limb loss. The behavioral system successfully detected significant differences between the groups using the narrow beam [27]. Likewise, adolescent idiopathic scoliosis patients performed walking tasks such as walking on the ground, on a line and on a beam more slowly than normal subjects [28].

A critical parameter in the protocol of the beam walking test is likely the number of trials. We applied 3 trials of our novel beam walking scoring system in mice. Consistently, it appeared that lower limb prosthesis users needed multiple trials of clinical beam walking to achieve stable performance [29]. Although our novel system was tested in the room having strong light, we will test if scores of beam walking are different in the room having dim light in near future. Likewise, the applicability of the system in rats will be assessed in the near future because rats are extensively used in experiments to explore therapeutic agents for SCI.

## Supporting information

S1 Fig Inter-rater reliability of the scores was assessed between two observers. The scores of both ICR and C57BL/6 mice 4 weeks after both mild and severe SCI (No. 1 mouse to No. 48 mouse in the left column) were given by observer 1 (Obs. 1, middle column) and observer 2 (Obs. 2, right column). The rate of concordance was estimated by kappa statistic.
(TIF)

S1 Video A representative SCI mouse which did not use hindlimbs for locomotion on the beam. The mouse got 0 point regarding position of hindlimbs in Fig 6A.
(MP4)

S2 Video A representative SCI mouse which sandwiched the beam with thighs. The mouse got 1 point regarding position of hindlimbs in Fig 6A.
(MP4)

S3 Video A representative SCI mouse which sandwiched the beam with plantar. The mouse got 2 points regarding position of hindlimbs in Fig 6A.
(MP4)

S4 Video A representative SCI mouse which put plantar on the beam. The mouse got 3 points regarding position of hindlimbs in Fig 6A. This mouse also shows valid steps with slips of a few times.
(MP4)

S5 Video A mouse showing slips on the beam. (MP4)
