# Supplementary figures and images for: A simple scoring of beam walking performance after spinal cord injury in mice
Source: PLoS One. 2022 Aug 11;17(8):e0272233. doi: 10.1371/journal.pone.0272233 (PMC9371269; doi:10.1371/journal.pone.0272233)

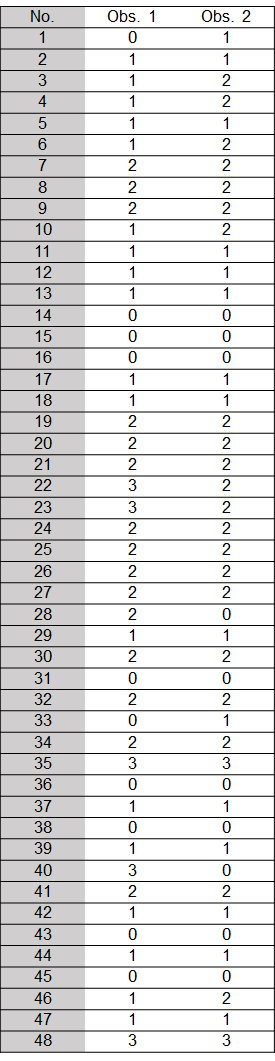

Supplement: S1 Fig — The scores of both ICR and C57BL/6 mice 4 weeks after both mild and severe SCI (No. 1 mouse to No. 48 mouse in the left column) were given by observer 1 (Obs. 1, middle column) and observer 2 (Obs. 2, right column). The rate of concordance was estimated by kappa statistic. (TIF) [file pone.0272233.s001.tif]
